# Unusual epileptic deterioration and extensive white matter lesion during treatment in Wilson’s disease

**DOI:** 10.1186/1471-2377-13-127

**Published:** 2013-09-25

**Authors:** Young Eun Kim, Ji Young Yun, Hui-Jun Yang, Han-Joon Kim, Beom S Jeon

**Affiliations:** 1 Department of Neurology, Seoul National University Bundang Hospital, Seoul National University College of Medicine, Gyeonggi, Korea; 2 Department of Neurology and Movement disorder center, Parkinson Study Group, Seoul National University Hospital, Seoul, Korea; 3 Department of Neurology, Ewha Womans University Mokdong Hospital, Seoul, Korea; 4 Department of Neurology, Ulsan University Hospital, Ulsan, Korea; 5 Department of Neurology, Seoul National University Hospital, Seoul National University College of Medicine, Seoul, Korea

**Keywords:** Wilson’s disease, Seizure, Dystonia, Cortical lesion: MRI

## Abstract

**Background:**

Wilson’s disease (WD) is a genetic disorder which can be controlled fairly well with decupuration therapy. However, symptoms, on rare occasions, can worsen even when WD is being treated. Herein, we report a case involving unusual neurological deterioration during decupuration therapy for WD.

**Case presentation:**

A 28-year-old man was diagnosed with WD 13 years prior to his clinical visit; however, his drug compliance has been poor over the years. He was treated with trientine because tremors and dysarthria have presented in recent years. However, dysarthria and dystonia developed in his limbs, which were worse on the right side and had been aggravated for several weeks despite good drug compliance. His symptoms were fluctuating. It was initially misdiagnosed as dystonia; although, it turned out to be a seizure due to cortical degeneration. These symptoms were completely resolved with antiepileptic drugs. Moreover, the cortical enhancement of bifrontal degeneration has disappeared on the MRI.

**Conclusion:**

This case showed unusual epileptic neurologic deterioration due to cortical degeneration during decupuration therapy. Seizures in WD can easily be mistaken as part of dystonia. However, the fluctuating symptoms suggest a seizure.

## Background

Wilson disease (WD) is a disorder involving copper metabolism characterized by impaired excretion of this metal [1]. Neurological worsening during decupuration therapy may be due to the side effects of the drug, improper treatment, poor drug compliance, drug resistance, or fulminant disease [2,3]. This case shows unusual neurological deterioration and cortical degeneration during decupuration therapy for WD.

## Case presentation

A 28-year-old male was diagnosed with WD in 1997, when he was 15 years old. Kayser-Fleischer ring was found incidentally during ophthalmological examination for itchy eyes. The serum ceruloplasmin was 3.5 mg/dl (normal range 15-40); the serum copper was 38 ug/dl (normal range 70-155); and the 24 hour urine copper was 1620 ug/day (normal range 0-75). Abdominal sonography showed chronic liver disease and brain MRI was compatible with WD; however, he did not have hepatic or neurological symptoms at that time. As the treatment regime, Penicillamine was tried initially, and then, it was switched to trientine because of the side effects such as paresthesia. Compliance was poor for 10 years because he did not have any symptoms. In late 2009, tremors and behavioral changes including irritability, aggressive behavior, and a somewhat depressive mood developed and became progressively worse. He visited our clinic in May 2010. Dystonic tremor in the right hand and mild dysarthria was the main feature upon initial examination. The serum ceruloplasmin was repeatedly less than 8 mg/dl; the serum copper was 37.3 ug/dl, and the 24 hour urine was 630 ug/day. Kayser-Fleischer ring was present. Trientine was prescribed with a gradual increase to 1000 mg; however, he complained of worsening stuttering, tremor, and gait disturbance for the next several months, and more aggravation in the 2 weeks prior to his admission. He attested he had good compliance in taking his meds.

He was admitted to the hospital to evaluate his deterioration in September 2010.

**Figure 1:**
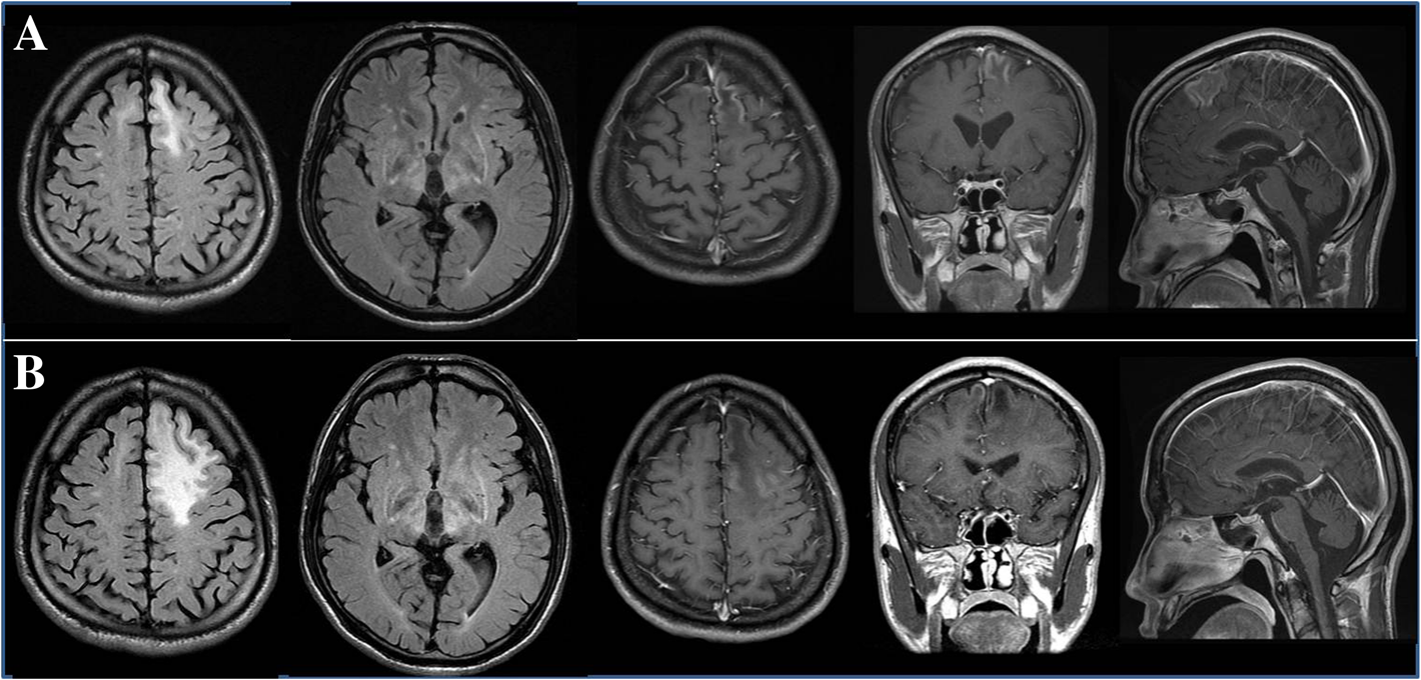
**The change of MRI over time (A) MRI before the treatment with antiepileptics after admission shows high signal intensity on both paramedian superior frontal gyri, both basal ganglia and thalamus in the FLARE image, and cortical enhancement on the left superior frontal gyrus in the T1 enhanced image. (B)** Cortical enhancement of left superior frontal gyrus resolved at 1 month after treatment with the antiepileptics.

Upon admission, he had dysarthria, stuttering, and bilateral dystonic movements in the limbs, which were worse on the right side. Neurological examination showed mild motor impairment, brisk deep tendon reflexes, and extensor plantar responses, mainly on the right leg. His speech disturbance and dystonia had peculiar fluctuations with intermittent worsening without change of alertness lasting from several minutes to an hour, and occurring many times a day (Additional file 1). At times, he became almost anarthric and unable to move the affected parts with stiffening. In general, his neurological condition progressively deteriorated with the above mentioned intermittent worsening during his hospital stay. Blood and urine studies showed good compliance for his medication (serum copper was 16.5 ug/dL and 24 hour urine copper was 613 ug/day). Brain MRI was done after we confirmed above neurologic findings and showed high signal intensity lesions on the bilateral paramedian superior frontal gyri as well as on the basal ganglia, thalamus, and brain stem on T2, Flare, and DWI. Contrast enhancement was present on the left superior frontal gyrus (see Figure 1A).

Video EEG monitoring showed multiple episodes of stiffening of the right arm followed by dystonic then rhythmic tremor of the right leg, which correlated with the left frontal rhythmic delta activity (Additional file 2). Levetiracetam 2500 mg and Trileptal 1200 mg markedly improved his neurological symptoms. Upon brain MRI, cortical enhancement almost disappeared one month after antiepileptic medications (see Figure 1B).

## Conclusions

This case presented unusual epileptic neurological deterioration in WD. Flutuating deterioration of focal neurologic deficit during decupuration can be mistaken as a part of dystonia in WD because dystonia is usually aggravated on action and it is rather common in WD. However, fluctuating symptoms suggested seizures in this case. Seizure should be considered as one of the causes of neurologic deterioration in WD.

The seizures are probably related to Wilsonian cortical damages in this case. The resolution of the neurological symptoms and cortical enhancement after antiepileptic medication suggests that there were secondary cortical changes following the seizures.

WD with extensive white matter lesions represents a rare neuropathological subgroup, the pathogenesis of which is not clearly determined [4-7]. These abnormalities occur mainly in the frontal lobe [5]. And most of the patients with extensive WML were non-treated patients with a very severe form of the disease, and had very poor prognosis [4]. Epilepsy is not a common manifestation of WD, but is present and more common in a WD population with WML rather than just the general WD population [6,7]. However there have been no reports on seizures provoking a secondary change of the WML in WD or on the resolution of the cortical enhancement following AED. Based on the chronological changes on MRI in our case, seizures appear to have contributed to extensive WML and cortical enhancement.

Epilepsy may mimic dystonia when the spells cause posturing. Nocturnal paroxysmal dystonia, which was previously misclassified as primary movement disorder, is now recognized to be a manifestation of nocturnal frontal lobe epilepsy [8]. De Silva et al. reported that prolonged hand spasm can be the only presentation of focal epilepticus [9]. Our case showed paroxysmal and fluctuating spasms, which prompted us to suspect epilepsy. Early suspicion and diagnosis of seizures can prevent the secondary generalization and complications.

## Consent

Written informed consent was obtained from the patient for publication of this Case report, any accompanying images and videos. A copy of the written consent is available for review by the Editor-in-Chief of this journal.

## Competing interests

All authors declare that they have no competing interests.

## Authors’ contributions

YEK: drafting/revising/critique the manuscript, study concept or design, collection of data. JYY: revising/critique the manuscript. HJY: revising/critique the manuscript. HJK: revising/critique the manuscript. BSJ: drafting/revising/critique the manuscript, study concept or design, supervision. All authors read and approved the final manuscript.

## Pre-publication history

The pre-publication history for this paper can be accessed here:

http://www.biomedcentral.com/1471-2377/13/127/prepub

## Supplementary Material

Additional file 1 **Segment 1 shows dysarthria and bilateral dystonic movements in the limbs, which are worse on the right side.** His speech disturbance and dystonia had peculiar fluctuations with intermittent worsening. Click here for file

Additional file 2 Segment 2 is a video EEG monitored segment showing an episode of the stiffening of the right arm followed by rhythmic tremor of the right leg, which correlated with the left frontal rhythmic delta activity and epileptiform discharges. Click here for file
